# Multimodality Imaging in Sarcomeric Hypertrophic Cardiomyopathy: Get It Right…on Time

**DOI:** 10.3390/life13010171

**Published:** 2023-01-06

**Authors:** Alessandro Galluzzo, Francesca Fiorelli, Valentina A. Rossi, Luca Monzo, Giulia Montrasio, Massimiliano Camilli, Geza Halasz, Giuseppe Uccello, Rocco Mollace, Matteo Beltrami

**Affiliations:** 1 Cardiology Unit, Santa Croce Hospital, 10024 Moncalieri, Italy; 2 Royal Brompton and Harefield Hospitals, Part of Guy’s and St. Thomas’ NHS Foundation Trust, London SW3 6NP, UK; 3 Department of Cardiology, University Hospital of Zurich, 8091 Zurich, Switzerland; 4 Centre d’Investigations Cliniques Plurithématique, Université de Lorraine INSERM, 54035 Nancy, France; 5 Inherited Cardiovascular Diseases Unit, Barts Heart Centre, St. Bartholomew’s Hospital, London EC1A 7BE, UK; 6 Department of Cardiovascular and Pulmonary Sciences, Catholic University of the Sacred Heart, 00168 Rome, Italy; 7 Department of Cardiovascular Medicine, Fondazione Policlinico Universitario A. Gemelli IRCCS, 00168 Rome, Italy; 8 Department of Cardiosciences, A.O. San Camillo-Forlanini, 00152 Rome, Italy; 9 Cardiology Unit, Medical Department, “Galeazzi—Sant’Ambrogio” Hospital, 20157 Milan, Italy; 10 Cardiovascular Department, Humanitas Gavazzeni, 24125 Bergamo, Italy; 11 Cardiology Unit, San Giovanni di Dio Hospital, 50143 Florence, Italy

**Keywords:** hypertrophic cardiomyopathy, phenocopies, left ventricular systolic dysfunction, left ventricular diastolic dysfunction, outcome, imaging

## Abstract

Hypertrophic cardiomyopathy (HCM) follows highly variable paradigms and disease-specific patterns of progression towards heart failure, arrhythmias and sudden cardiac death. Therefore, a generalized standard approach, shared with other cardiomyopathies, can be misleading in this setting. A multimodality imaging approach facilitates differential diagnosis of phenocopies and improves clinical and therapeutic management of the disease. However, only a profound knowledge of the progression patterns, including clinical features and imaging data, enables an appropriate use of all these resources in clinical practice. Combinations of various imaging tools and novel techniques of artificial intelligence have a potentially relevant role in diagnosis, clinical management and definition of prognosis. Nonetheless, several barriers persist such as unclear appropriate timing of imaging or universal standardization of measures and normal reference limits. This review provides an overview of the current knowledge on multimodality imaging and potentialities of novel tools, including artificial intelligence, in the management of patients with sarcomeric HCM, highlighting the importance of specific “red alerts” to understand the phenotype–genotype linkage.

## 1. Introduction

Hypertrophic cardiomyopathy (HCM) is the most common inheritable cardiomyopathy with autosomal dominant transmission, and it is characterized by heterogeneous presentation and outcome [1]. The assessment of left ventricular (LV) systolic function provides essential information in terms of prognosis and risk stratification. However, unlike other cardiomyopathies, the only use of the LV ejection fraction (EF) for this purpose may lead to underestimation of the disease severity, because it remains relatively preserved even at more advanced stages. Moreover, HCM is universally defined as a ‘diastolic disease’ and assessment of LV diastole is another key element for clinical management and outcome prediction. Unfortunately, the standard approach that estimates the degree of systo-diastolic dysfunction in other cardiomyopathies could be misleading when applied in this setting. In recent years, guidelines and position papers have been developed to guide morphologic and functional assessment of systole and diastole in HCM: from the LVEF to the global longitudinal strain (GLS) by speckle tracking echocardiography, from the mitral inflow pattern to tissue Doppler imaging (TDI) images and tricuspid velocity [2,3,4,5].

In addition, cardiac hypertrophy can be the manifestation of inherited and acquired systemic disorders with different genetic background, prognosis, and treatment. Multimodality imaging can be useful in differentiating phenocopies and identifying specific red flags.

Although multiple imaging modalities are available, an integrated approach is yet to be defined.

The aim of this review is to provide a wide overview of the current knowledge and future frontiers in multimodality imaging for HCM, highlighting the potentialities of different techniques in the management of this cardiomyopathy. The literature research methods adopted are described in Supplementary File S1.

## 2. An Overview of the Main Imaging Techniques in Hypertrophic Cardiomyopathy

### 2.1. Echocardiography

Transthoracic echocardiography (TTE) is the initial and preferred imaging modality to confirm diagnosis, stratify prognosis and family screening [5]. TTE enables assessing the severity and pattern of LV hypertrophy (LVH), possible obstruction in the LV outflow tract (LVOTO) and mitral valve pathology.

#### 2.1.1. Definition and Patterns of Hypertrophy

**Figure 1:**
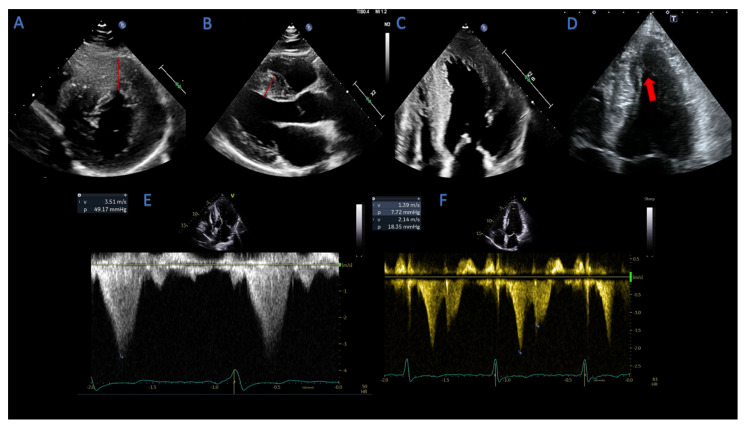
Echocardiographic patterns of hypertrophic cardiomyopathy. (**A**) focal asymmetric anterior wall hypertrophy (red line), parasternal short axis view; (**B**) focal asymmetric septal hypertrophy (red line), parasternal long axis view; (**C**) concentric hypertrophy pattern; (**D**) apical hypertrophy pattern (arrow); (**E**) dagger-shaped profile of CW doppler in LVOTO; (**F**) lobster claw profile of CW doppler in mid cavity obstruction.

HCM is defined by the presence of an end diastolic wall thickness ≥15 mm in any LV segment, in the absence of abnormal loading conditions; however, a wall thickness ≥13 mm is diagnostic in case of family history of HCM or in disease-causing gene carriers [2,6]. HCM can present with different phenotypes and patterns of LVH, and an accurate evaluation of LVH patterns is essential for patients’ management and outcomes’ prediction [2,3]. The most common pattern of hypertrophy in HCM is the focal asymmetric hypertrophy of the basal anterior septum, defined as a ratio of septal/posterior wall thickness >1.3 in normotensive individuals, and >1.5 in hypertensive patients (Figure 1A,B). Other variants include a sigmoid septum (which poses challenges in the differential diagnosis with hypertensive heart disease), reversed septal curvature, concentric LVH (Figure 1C), mid-wall, and apical hypertrophy (Figure 1D). More subtle anatomical variants, such as myocardial crypts (narrow, deep blood-filled invaginations within LV myocardium) and apical displacement of the papillary muscles, can precede overt LV hypertrophy in pathogenic genes carriers [6]. Occasionally, hypertrophy can involve the right ventricle (RV), and ideally, RV hypertrophy (RVH) should be assessed at the level of the RV free wall in zoomed subcostal views in tele-diastole [5]. Ultrasound enhancing agents should be used whenever the image quality is suboptimal, particularly to better visualize the apical region, as well as the eventual presence of apical aneurysms and ventricular thrombi.

#### 2.1.2. Systolic Function

Myocardial hypertrophy and fibrosis are the main determinants of global and regional abnormalities in LV myocardial mechanics and contribute to systolic dysfunction and LV remodeling. Due to increased wall thickness with a smaller LV cavity, HCM patients show a reduced stroke volume index, longer LV isovolumetric contractions and ejection times, with a delayed peak systolic velocity [7]. The LV ejection fraction (EF) ranges from normal to hyperdynamic (55 to >70%), whereas an EF < 50% is indicative of LV dysfunction and correlates with higher rates of adverse events, including all-cause mortality and cardiac transplantation [8]. However, the wall thickness typically determines small cavities with the normal or supernormal LVEF, despite a progressive reduction in myocardial shortening function, whereas a decrease in the LVEF is limited to late advanced stages. Considering the persistence of normal LVEF values throughout most of HCM natural history, this parameter provides inadequate metrics of LV systolic performance, whilst global and regional longitudinal deformation better expresses how this disease affects systolic myocardial mechanics. An impairment of the LV global longitudinal strain (GLS), particularly in the basal-septal segments, has been described in patients with asymmetrical HCM and normal EF [9]. Although the extent of strain abnormalities and their regional expressions vary according to the degree of hypertrophy, they may also be pathological in segments with relatively normal wall thickness, most likely due to underlying disarray or fibrosis. Given the patchy distribution of fibrosis in HCM, as explained below in Section 2.3, a segmental analysis of the LV longitudinal strain may be even more accurate in assessing fibrosis than a global evaluation: in a cohort of 46 HCM patients, Wabich et al. recently demonstrated that segmental, rather than the GLS, with a cut-off value of −12.5%, has a higher sensitivity for the identification of LGE on CMR, and can help identify patients who need CMR referral for better risk stratification [10].

Previous observations have suggested that the reduction in the GLS is counterbalanced by an increase in the global circumferential strain (GCS), resulting in a biplanar strain vector more circumferentially orientated and in a normal LVEF [11]. Analyses of the 3D strain seem to confirm a reduction in the 3D-GLS with a complementary increase in the GCS in segments without (or with only mild) hypertrophy, and a reduction only in severely hypertrophied regions [12]. Other authors described the lower mid- and epicardial GCS in HCM patients, suggesting that LVEF preservation could be the result of the raised endocardial GCS with a compensatory increase in radial strain [13].

Moreover, an increased base-to-apex gradient rotation with a higher degree of twist displaced to the apex has been observed [11]. Accordingly, data obtained by the 3D strain show an increase in peak LV twist, predominantly due to an increased apical rotation [14].

Overall, analyses of strain-derived longitudinal and circumferential mechanics, LV torsion and twist give valuable information to better understand the underlying pathophysiology of HCM. In addition, these parameters may help in early identification of structural changes in genotype-positive, phenotype-negative patients.

Data on myocardial mechanics may also help in risk stratification and therapeutic management during follow-up: on top of the conventional sudden cardiac death (SCD) risk factors, a reduction in the GLS, a marker of myocardial fibrosis, was shown to be an independent predictor of appropriate ICD therapy [15]. Although it is challenging to identify a prognostic cut-off value of the GLS, it has been shown that a reduction below −16% represents an echocardiographic independent predictor for heart failure hospitalization, sustained ventricular arrhythmias, all-cause death [16], as well as conferring a higher probability of AF occurrence [17].

#### 2.1.3. Diastolic Function

Diastolic dysfunction is the hallmark of the disease. It is a common finding in symptomatic HCM patients and is due to impaired LV relaxation, as a consequence of increased myocardial stiffness, and impaired left atrial (LA) function [5]. Although cardiac catheterization remains the gold standard to directly measure LV filling pressures, echocardiographic assessment of diastolic function is largely widespread [18]. The assessment of diastolic function in HCM includes (1) mitral inflow velocities recorded at the leaflet tip level, (2) septal and lateral early diastolic velocity of the mitral annulus by tissue Doppler, (3) peak tricuspid regurgitation (TR) velocity obtained by continuous wave (CW) Doppler, (4) biplane LA maximum volume index (LAVi), 5) and pulmonary vein velocities. The presence of at least 2 abnormal values out of 3 (LAVi > 34 mL/m^2^, average E/e’ ratio > 14 and a peak TR velocity > 2.8 m/s) is indicative of elevated LA pressures. In the presence of significant MR (≥moderate), peak TR velocity and pulmonary vein atrial reversal velocity are the most reliable indexes of LV filling pressures [5,18]. A restrictive LV filling pattern (E/A > 2, with increased E/e’ ratio > 14) in HCM patients is associated with HF hospitalizations, reduced exercise tolerance, and SCD [5,18,19].

While these metrics are not accurate in all clinical scenarios [18], recent studies revealed that some strain-derived parameters can help in assessing diastolic dysfunction [20]. Garceau et al. investigated the correlation between invasive hemodynamic parameters obtained with cardiac catheterization, with 2D-strain imaging in patients with HCM [21]. The main finding of their study was that the LV end diastolic pressure correlates with the fraction of early apical reverse rotation, a measure of apical decrease in the rotation angle from its peak value, which is reduced in HCM.

LA remodeling results from left ventricular stiffness, LVOTO, and MR, but also from primary atrial involvement in the myopathic process (the so-called “atrial cardiomyopathy”). Impairment of LA function is an important contributor to diastolic dysfunction in HCM and can be assessed by the LA strain [5,22]: a recent study evaluating LA volumes and the 3D strain confirms a reduction in global and mean segmental strain parameters in HCM patients as compared to controls [23]. Abnormal LA function is also associated with symptoms severity, exercise intolerance and adverse cardiovascular outcomes [22]. Iio et al. [24] found a reduction in the peak negative global strain during atrial contraction and the peak positive strain during ventricular systole in HCM, which correlated with an increased risk of atrial fibrillation (AF) and cardiac events. In a recent HCM cohort, the 2D LA strain below 22% (together with values of the LV GLS below −16%) allowed for AF prediction with high probability [17]. Furthermore, a decrease in the peak LA strain and LA dilatation is associated with increased risk of stroke even in patients without clinical arrhythmias [22,25]. However, LA strain applicability in clinical practice, as guidance for oral anticoagulation in patients with HCM, requires further investigation.

Additionally, this technique was further used in family members of HCM patients for early detection of myocardial changes, especially in pathogenic mutation carriers [26]: The LA minimum volume and a lower peak atrial longitudinal strain were observed in mutations carriers compared to controls. These data show how genotype-positive subjects may be distinguished from healthy controls through detection of LA abnormalities [27]: although only a few studies have explored atrial function in HCM, strain abnormalities could reflect subtle structural changes in LA wall due to intrinsic atrial myopathy and diastolic dysfunction, not clearly detectable in the early stages of HCM.

#### 2.1.4. Assessment of LVOT- and Mid-Cavity Obstruction

LVOTO occurs in up to two-thirds of HCM patients [2,3,5]. Resting LVOTO is considered significant, with a peak gradient ≥30 mmHg, as this is associated with increased risk of SCD and progression of HF symptoms [28]. However, LVOTO is a dynamic phenomenon that can largely vary within the same patient, depending on afterload, preload, and LV contractility. The main anatomical abnormalities that contribute to LVOTO are septal hypertrophy, although the systolic anterior motion (SAM) of mitral leaflet, antero-apical displacement of the papillary muscles and chordal slack can contribute to this phenomenon.

Traditionally, the M-mode has been used to assess the anterior mitral leaflet–septal contact, with severe LVOTO defined as contact for ≥30% of systole, and to depict mid-systolic closure (“notching”) of the aortic valve, caused by a drop in subaortic pressure [29]. Color Doppler is used to localize turbulent flow or aliasing, which indicates increased velocity. Precise localization of obstruction is detected using pulsed-wave (PW) Doppler in the apical 5- or 3-chamber view. Maximal peak LVOT gradient is calculated measuring the peak LVOT velocity with CW Doppler and derived from the simplified Bernoulli equation [5]. Typically, in LVOTO, velocities increase slowly in early systole, then rise abruptly and peak in mid-to-late systole, resulting in a characteristic “dagger-shaped” profile (Figure 1E), different from the Doppler spectral profile of valvular aortic stenosis. In cases of relevant MR, distinguishing high-velocity LVOT flow from MR flow may become challenging due to anatomical proximity of the two flows. However, the MR envelope usually peaks earlier than the one of the LVOT flow, is more rounded, and the velocity is always higher than LVOT velocity (>5–6 m/s) [5].

Resting or provoked gradients of ≥50 mmHg are considered a threshold for invasive therapy in patients who have refractory symptoms, despite maximally tolerated dose of medical therapy [2,3]. TTE and, in selected cases, TOE play an important role in patient selection for septal reduction therapies, determining the precise location of the dynamic obstruction as well as the presence of coexisting mitral valve or papillary muscle abnormalities, that would portend for surgical myectomy rather than alcohol septal ablation. Aortic stenosis and subaortic membranes should be ruled out with TTE +/− TOE prior to intervention.

Less frequently, echocardiography may identify mid-cavity obstruction, which can coexist with LVOTO or represent the main obstruction pattern, promoting the formation and growth of apical aneurysms [30]. In mid-cavity obstruction, a typical “lobster claw” profile in the CW Doppler envelope can be seen, with a mid-systolic abrupt cessation of midventricular flow due to a sudden rise in afterload (Figure 1F). The absence of flow during mid-cavity obstruction renders the pressure gradient inaccessible to Doppler measurement; hence, peak Doppler gradients may be lower than those obtained with cardiac catheterization. In such cases, an early diastolic or late systolic jet from the apical chamber to the LV base may be seen, representing the emptying of an apical cavity at the onset of diastole [5].

#### 2.1.5. Mitral Valve Abnormalities in HCM Patients

Many patients with obstructive HCM have abnormalities of the mitral valve and the subvalvular apparatus, which contribute to SAM. In fact, SAM is a result of the Venturi effect on elongated mitral valve leaflets, defined as an anterior mitral leaflet >2.5 cm (>1.6 mm/m^2^) and/or a posterior leaflet >1.5 cm [5,31]. The mitral regurgitation (MR) related to SAM of the mitral valve is typically posteriorly oriented. In this case, septal myectomy alone is adequate to treat the MR and a valve intervention is not recommended [2,5]. In these cases, due to the eccentricity of the regurgitant jet, quantitative assessment using the proximal isovelocity surface area (PISA) method can lead to erroneous estimation of MR severity [5]. However, the presence of a central or an anteriorly directed jet should prompt careful evaluation, potentially with TOE, as MR related to intrinsic mitral valve disease (such as mitral valve prolapse, chordal elongation or rupture with flail) not infrequently occurs in HCM patients and must be addressed separately [32].

### 2.2. Stress Echocardiography

Stress echocardiography plays an important role in both the diagnostic process and prognostic stratification of HCM patient: this test has now become a gold standard in clinical practice to measure dynamic LV outflow gradient provoked by exercise [2].

In all patients undergoing rest echocardiography, a Valsalva maneuver should be performed: unfortunately, its sensibility is low and its efficacy can be variable due to patients’ compliance [33]. Thus, if bedside maneuvers fail to induce LVOTO > 50 mmHg, exercise stress echo is the most physiological instrument to evoke it and can be performed with treadmill or bicycle depending on the laboratory experience. Provoked gradients detection directly influences therapeutic choices: patients with significant gradients during exercise and HF symptoms despite optimal medical therapy candidate for surgical myectomy or alcoholic septal ablation. Several studies have shown how exercise echocardiography provides different prognostic information too: exercise-induced LVOTO is associated with advanced and drug-refractory HF symptoms [34] and new-wall motion abnormalities or a reduced coronary flow reserve (≤2) on left anterior descending coronary artery are related to a composite endpoint of death, arrythmias, heart transplantation and acute HF [35]. Furthermore, data on functional capacity and exercise tolerance can be obtained: the onset of LVOTO in the early phase of exercise has been associated with a worse NYHA functional class, while gradients occurring at the peak of exercise or immediately thereafter are associated with better prognosis and NYHA class [36]. In addition, exercise echocardiography provides important information regarding diastolic reserve. HCM patients have lower mitral annular early diastolic (e’) and systolic (s’) velocities at rest and a diminished increase in exercise testing [37,38]; a failure to increase diastolic reserve during exercise has been associated with reduced exercise duration (<500 s) [38]. Exercise-induced LV diastolic dysfunction could explain exertional dyspnea and increased exercise intolerance: despite technical issues, the diastolic function under stress should be reported in all patients [39].

In recent years, additional data were provided by speckle-tracking strain analysis, showing that the post-stress strain rate correlates with exercise capacity: in one study on ninety-five HCM patients, obstructive and non-obstructive HCM patients, compared with labile-obstructive patients and controls, showed reduced mechanical reserve (post-exercise percent change in the systolic strain rate) [40]. Other speckle-tracking stress parameters, such as left ventricular desynchrony and attenuated systolic functional reserve, were impaired in HCM patients compared with normal subjects, and with patients affected by systemic hypertension with left ventricular hypertrophy [41]; untwist rates are related to delayed left ventricular filling with exercise, and decreased exercise tolerance in patients with non-obstructive HCM [42].

Stress echocardiography may improve SCD risk prediction also in case of hypotensive response to exercise or a reduced increase in blood pressure (<20 mmHg) compared to basal values [43]. Moreover, lower exercise strain rates and mechanical reserves during exercise echocardiography are independent predictors of ventricular tachycardia and ventricular fibrillation; these could identify patients at risk of SCD, as demonstrated by Pozios and colleagues [40].

Finally, a classical application of exercise echocardiography, as the detection of myocardial ischemia, can be useful in hypertrophic cardiomyopathy. Chest pain and myocardial ischemia are common in HCM patients and have multiple underlying mechanisms (e.g., stenosis of coronary vessels due to increased myocardial thickness, increased oxygen demand and intraventricular pressures or decreased capillary density). Although LV wall motion abnormalities are frequent in this setting [44], the presence of coronary artery disease carries an increased risk of death in HCM patients [45]. In addition left ventricular asynergy detection during stress echocardiography represents an independent predictive factor for cardiac-related death and serious events, such as cardiac transplantation [46].

### 2.3. Cardiovascular Magnetic Resonance

Cardiovascular Magnetic Resonance (CMR) represents the most accurate imaging modality to define and differentiate the spectrum of myocardial disease, with higher diagnostic sensitivity than echocardiography. Despite being the first imaging diagnostic approach for patients with suspected HCM, echocardiography has several limitations caused by poor acoustic windows, particularly for segments not well visualized by ultrasound [4]. Several studies have demonstrated a discrepancy in measurements of LVWT between echo and CMR [47]: CMR has considerably increased the detection of circumscribed areas involved by focal hypertrophy, such as apical myocardium and mid portions of anterior and inferior interventricular septum, frequently underestimated by echo analysis [48]. Conversely, echocardiography tends to overestimate LVWT due to the inappropriate inclusion of papillary muscle or RV trabeculation as a consequence of image plane obliquity to obtain parasternal long axis windows [47]. Thanks to its high spatial resolution and its exceptional capacity to differentiate the interface between myocardium and blood pool, CMR plays a crucial role especially in the early stages of the disease. In addition, morphological features including mitral valve elongated leaflets, myocardial crypts or an LV apical–basal muscle bundle may uncover preclinical stages of HCM [49,50,51].

What makes CMR a unique imaging modality is the opportunity of tissue characterization. Late gadolinium-enhanced cardiac magnetic resonance (LGE-CMR) represents the gold standard imaging method to assess myocardial fibrosis. Fibrosis detection in HCM plays a crucial role in risk stratification, being a strong and independent predictor of several complications of HCM and correlating with HF symptoms and advanced stages [52]. A reduction in LVWT is strictly related to the presence of LV fibrosis, it represents a marker of disease progression and portends a worse prognosis. Several studies have shown a significant correlation between LGE extension and arrhythmic burden in HCM; therefore, LGE-CMR provides an additional independent predictive value to current protocols of SCD risk [53]. In fact, HCM patients at high risk for SCD have shown a considerably higher extension of LGE compared to low-risk patients, and this risk progressively increases across the range of LGE amounts, with LGE ≥ 15% of LV mass conferring a >2-fold increased risk [54].

However, the myocardium in HCM is typically characterized by many forms of tissue remodeling [55]. Interstitial fibrosis, due to expansion of extracellular volume and deposition of extracellular matrix, probably represents the most common process of tissue modification in HCM [56]. Replacement fibrosis, conversely, is secondary to microvascular remodeling and failure of intramural arterioles, and it is expressed by the typical patchy pattern detected by LGE-CMR at the level of hypertrophied segments [57]. While LGE-CMR has a high sensitivity to detect circumscribed regions of replacement fibrosis, the limited spatial resolution does not permit an optimal assessment of local interstitial expansion [58]. The detection of diffuse interstitial fibrosis, which anticipates the phenotypic manifestations of the disease, may aid in HCM staging.

Particularly in this setting, T1 mapping represents a valuable tool in HCM. T1 mapping is a CMR technique that enables the identification of tissue abnormalities, including interstitial fibrosis, oedema and amyloid, through quantification of longitudinal relaxation time. T1 time varies in correlation with the state of the molecular environment, and is susceptible to pathological processes that modify tissue water composition [59]. It represents an innovative tool in the identification of tissue modifications in HCM patients even in the absence of abnormalities identified by traditional post-contrast sequences. Indeed, an increase in T1 and T2 values in myocardial segments with normal wall thickness has been reported in patients affected by HCM, as a confirmation that tissue remodeling may precede morphological and functional abnormalities [60]. Furthermore, Kato et al. detected increased native T1 values in more than 30% of myocardial segments without LGE, highlighting a strong correlation between thickening and native T1 that, contrarily to LGE, is able to identify diffuse interstitial fibrosis [61]. Modifications of myocardial T1 relaxation time before and after contrast injection, as compared with the modification of T1 relaxation of blood, allow for an estimate of interstitial space through a quantification of extracellular volume (ECV) [62]. Many studies demonstrated a positive relationship between ECV and HCM, especially when fibrosis deposition is related to a predominant expansion of interstitial space [63].

Challenges in CMR for HCM include the variability in quantification techniques, lack of standardized approaches and cut-offs, as well as limited knowledge of LGE progression patterns.

## 3. A Practical Approach to Multimodality Imaging in HCM

### 3.1. Differential Diagnosis of Phenocopies

**Figure 2:**
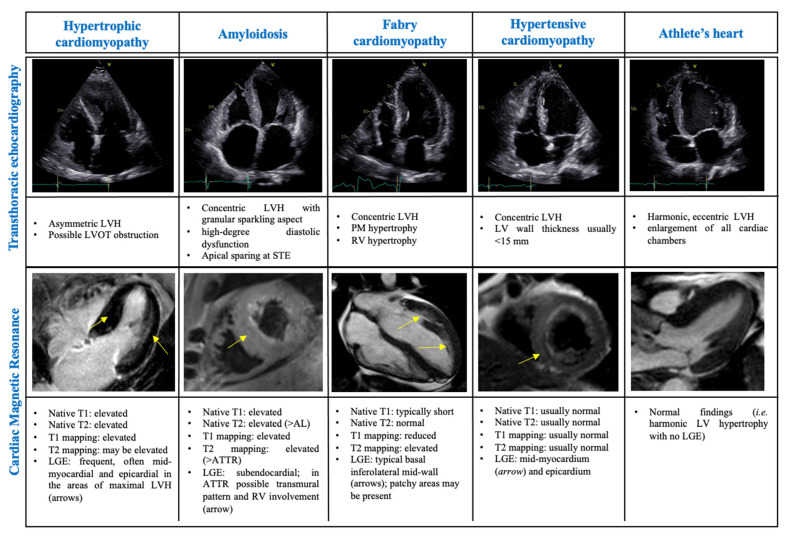
Echocardiographic and cardiac magnetic resonance ‘red flag features’ for the diagnosis of hypertrophic cardiomyopathy and phenocopies. Abbreviations: AL, light-chain amyloidosis; ATTR, transthyretin amyloidosis; LGE, late gadolinium enhancement; LV, left ventricle; LVH, left ventricular hypertrophy; LVOT, left ventricular outflow tract; RV, right ventricle, STE, speckle tracking echocardiography.

Although LV hypertrophy (LVH) is a key feature in sarcomeric HCM, several other cardiac, systemic or metabolic diseases mimic the hypertrophic phenotype. Given that the management and prognosis of different aetiologias of LVH may significantly differ, an accurate diagnosis is crucial (Figure 2).

#### 3.1.1. The Athlete’s Heart

The distinction between hypertrophic cardiomyopathy and the athlete’s heart is a common clinical dilemma, since LVH is a key feature of both phenotypes. The athlete’s heart is defined by structural, electrical and functional adaptation to repetitive, high-intensive or extensive exercise [64]. In particular, isometric, static exercise with repetitive pressure overload results in a biventricular, typically balanced, myocardial hypertrophy [65].

Up to 2% of athletes may develop a borderline LVWT between 13 and 15 mm [64,66]. As such, several efforts have been made to define adequate cut-off of LVWT based on TTE to distinguish it from a mild HCM phenotype. Echocardiography plays a fundamental role as screening tool to differentiate between a physiological, training-related cardiac remodeling from pathological cardiac remodeling seen in HCM [67]. Compared to pathological alterations, exercise-induced chamber thickening is not accompanied by loss of function, is reversible after detraining and usually present with a harmonic and symmetric hypertrophy of all cardiac chambers [68]. Further echocardiographic features include different kind of hypertrophy, which is typically eccentric in athletes and concentric in HCM, and generally the presence, in HCM, of reduced LV function and reduced global LV strain analyses, diastolic dysfunction with increased LV filling pressures and reduced e` velocity, reduced atrial function, and LA deformation indexes [66,69].

CMR further helps in distinguishing between physiological remodeling of the athlete’s heart from HCM: in the latter, LGE is often present in contrast to the athlete’s heart and is a well-validated sign of focal myocardial lesions and fibrosis due to pathogenic HCM-related sarcomeric alterations [2,70].

#### 3.1.2. Amyloidosis

Amyloidosis is a restrictive infiltrative cardiomyopathy characterized by deposition of amyloidogenic, misfolded insoluble proteins—mostly wild-type or inherited mutated transthyretin (ATTR) and light chains (AL)—in the extracellular matrix between myocytes [71]. This accumulation results in a relevant increase in cardiac wall thickness in the absence of an actual cardiomyocyte hypertrophy, which makes the differential diagnosis with HCM challenging [72]. Both hypertrophic phenotypes are characterized by structural morphologic alterations, leading primarily to high-degree diastolic dysfunction with increased LV filling pressures, hypertrophic ventricular walls with a concentric pattern, atrial septal thickening and reduced the left and right ventricular longitudinal strain [73]. Moreover, cardiac amyloidosis is characterized by a typical granular sparkling aspect of the hypertrophied myocardium on echocardiography due to the increased echogenicity derived from amyloid fibril deposits. However, this feature is not pathognomonic nor specific. CMR provides a better morphologic definition and is characterized by LGE with non-coronary distribution both within the ventricular myocardium and in the atria [74]. LGE usually present with a subendocardial pattern, although in ATTR a predominant transmural pattern as well as an involvement of the right ventricle has been described [75]. However, the gold-standard for diagnosis of cardiac amyloidosis remains an endomyocardial biopsy demonstrating amyloid deposits after Congo red staining, allowing its exact definition with mass spectrometry, immunohistochemistry or immunoelectron microscopy [76].

Given the high affinity of transtiretin for bone tracers, single-photon emission computerized tomography (SPECT) with bisphosphonates has emerged as a crucial tool for diagnosing transtiretin amyloidosis: specific diagnostic algorithms, that integrate bone SPECT, hematologic tests and imaging with ETT and CMR, have been proposed [76].

#### 3.1.3. Hypertensive Cardiomyopathy

Systemic arterial hypertension is highly prevalent and is a leading cause of cardiovascular morbidity and mortality. Since both HCM and hypertensive heart disease are characterized by an increase in LV mass, differentiation of these two phenotypes can be challenging, also due to their considerable morphological overlap. Increased LV mass, determined by echocardiography, is present in 23% to 48% of hypertensive patients [77]. The degree of hypertrophy is influenced by several variables such as ethnicity, neuro-humoral factors, and genetic variants [78]. In general, in HCM, LV wall thickening is usually asymmetrical (LV segments do not demonstrate similar thicknesses), or characterized by non-contiguous areas of focal hypertrophy (“lumpy” hypertrophic pattern), while hypertensive heart disease most often produces more symmetrical (or concentric) patterns [79]. Strain imaging, although not conclusive, may also be helpful to distinguish these two entities [80]. Other morphologic features, such as LV wall thickness ≥15 mm, abnormalities in papillary muscle morphology, LV apical aneurysm, RV hypertrophy, myocardial crypts and advanced diastolic dysfunction can favor the HCM phenotype vs. hypertensive heart disease [27,79]. These two conditions can also coexist, showing a pattern characterized by greater LV mass, more severe LV hypertrophy, and more abnormal GLS compared to non-hypertensive HCM [27]. In particular, the GLS is reduced in both HCM and hypertensive cardiomyopathy; the former is characterized by lower GLS absolute values, with higher endo-epicardial ratio, especially in hypertrophic segments, while the latter shows an equally distributed reduction in the GLS in all LV segments [13]. A systolic strain cut-off value of −10.6% has been previously shown to discriminate between HCM and H-LVH with a predictive accuracy of 91.2%, and even higher when combined with the septum/posterior wall thickness ratio [80]. Moreover, left ventricular desynchrony, expressed by time interval from the R-wave to peak radial or circumferential strain, appears more pronounced in HCM than hypertensive heart disease [81].

Resting or exercise-induced LVOTO can be observed also in hypertension (although rarely) and does not constitute a diagnostic criteria [82].

Although LGE may be present in the mid-myocardium and epicardium also in hypertensive patients, in HCM, it is typically located in the areas of maximal hypertrophy and at the insertion of RV into ventricular septum [83]. An increase in diffuse myocardial fibrosis, assessed by CMR T1 mapping, was recently demonstrated as a promising technique for discrimination of HCM from hypertensive heart disease [84].

#### 3.1.4. Fabry Cardiomyopathy

Fabry disease is an X-linked recessive lysosomal storage disorder that causes progressive myocardial accumulation of glycoshpingolipids, leading to LV hypertrophy, myocardial fibrosis and HF [85].

Morphological imaging assessment in Fabry cardiomyopathy may display concentric LV hypertrophy, disproportionate hypertrophy of papillary muscles, loss of base-to-apex circumferential strain gradient, and RV hypertrophy with normal systolic function, although none of them is a pathognomonic feature [86]. Asymmetric septal hypertrophy and even LVOT obstruction may also occur, making the clinical presentation similar to the sarcomeric HCM. Tissue characterization by CMR is therefore crucial for further differentiation. Typical LGE pattern of basal inferolateral mid-wall is seen in ~50% of cases [87], although other cardiac conditions (e.g., myocarditis, Chagas disease, sarcoidosis) may show a similar feature. T1 mapping plays a prominent role in the diagnosis of Fabry cardiomyopathy [88]. Indeed, glycosphingolipid infiltration significantly shortens myocardial T1 relaxation times, allowing to differentiate Fabry cardiomyopathy from other pathogeneses with increased wall thickness irrespective of sex and LV morphology and function [89]. In contrast, extracellular volume is typically normal, except for LGE-positive areas.

Novel echocardiography techniques may increase the diagnostic capacity in this setting. In fact, a recent study showed an independent and incremental value of RV speckle-tracking analysis in discriminating Fabry cardiomyopathy from HCM, above conventional echocardiographic ‘red flags’ [90].

In summary, echocardiography and CMR represent the main imaging tools for the differential diagnosis of HCM from its phenocopies. In particular, LV and RV speckle-tracking analysis and the GLS represent novel promising techniques [53,56,67,77]. Furthermore, echocardiography provides important parameters for prognostic stratification (including the morphologic analysis of LV septum thickness, LVOT flow characterization and the presence of apical aneurysm) [17,66,68]. CMR, by providing data on tissue characterization, has a high specificity for excluding phenocopies and it should be considered a routine part of HCM patient evaluation [91].

Among new imaging techniques, cardiac positron emission tomography (PET) has been proposed to quantify the myocardial blood flow in HCM. Impaired myocardial flow reserve is directly related with myocardial fiber disarray and hypertrophy in HCM and may lead to myocardial ischemia and ventricular arrhythmias [92]. Although this finding is not specific for HCM, PET has lately been proposed as a further risk stratification tool in selected patients [93].

### 3.2. The Appropriate Exam at the Right Time: Indications for Imaging Referral

#### 3.2.1. Imaging in “Non-Hypertrophic” Phase and “Classic Phenotype” HCM

In the “non-hypertrophic” phase, regular follow-up with imaging is used for screening of early phenotypical expression. However, due to incomplete penetrance and age-related onset, genotype-positive individuals may not phenotypically express the disease until the 7th decade. Even if the main diagnostic criterion for HCM is myocardial hypertrophy, several other functional and morphological abnormalities may be detected, as previously described in this review. None of them is essential for the diagnosis or adds information on prognosis, but their presence may lead to a closer follow-up. Current European recommendations suggest TTE in first-degree relatives as part of the initial family screening [2,4]. Afterwards, echocardiography is recommended once every 1–2 years between 10 and 20 years of age, and every 2–5 years in adults. CMR should be performed in case of suboptimal or borderline echo results, high-risk cases when the diagnosis of HCM would have direct implications on management, or when electrocardiography (ECG) and TTE provide conflicting results. The recent American guidelines on Hypertrophic Cardiomyopathy have similar indications for echocardiography [3]; however, they suggest longer screening intervals: 2–3 years for children and adolescents without genotype-positive or early-onset families; 3–5 years for adults.

Stable phases in HCM are defined as “classic phenotype” stages [94]. These represent the typical condition of the stable phenotype-positive sarcomeric hypertrophic heart, with or without LVOTO and various degree of diastolic dysfunction. At this stage, multimodality imaging is useful for phenotype characterization and monitoring possible disease progression. In addition, some features (e.g., LA dilatation, LV hypertrophy magnitude and LVOTO) correlate with SCD and are used for risk stratification and indication of primary prevention implantable cardioverter-defibrillators (ICDs).

Patients with HCM and LVOTO or AF are more symptomatic for heart failure; therefore, yearly TTE should be performed.

Exercise echocardiography is a routine test in patients with HCM and should be repeated every 6 months in the obstructive phenotype and every two years in all the other HCM patients, to screen for indication for myectomy or pharmacological therapy such as mavacamten.

CMR is recommended by the ESC guidelines during the initial diagnostic work-up in all patients with HCM and repeated every 5 years afterwards. The LGE extent and distribution identify the typical sarcomeric form and strongly correlates with prognosis. In fact, a repeated scan between 3 and 5 years is recommended by the American Heart Association to better predict SCD. Although not included in the HCM Risk-SCD score, the greater importance of the LGE extent and apical aneurisms is acknowledged in the latest ESC guidelines on ventricular arrhythmias and SCD [95,96,97]. In addition, CMR can provide anatomical details for patients referred for surgical treatment, such as extension of hypertrophy, the presence of mitral valve abnormalities, accessory chordae/papillary muscles and recognized apical aneurysms and thrombi [31].

#### 3.2.2. Imaging in HCM “Adverse Remodeling and Overt Dysfunction”

Fifteen to twenty percent of patients with HCM present an unfavorable clinical profile, with slow and progressive adverse ventricular remodeling. Overt LV dysfunction occurs in 5–10% of cases with two different morphofunctional patterns: the “hypokinetic-dilated” variant, characterized by volume increase and LVEF impairment (<50%); and the “hypokinetic-restrictive” variant, characterized by a small and stiff LV, with severe diastolic dysfunction, regardless of any systolic function deterioration. In patients with “adverse remodeling” and “overt dysfunction” phase, the outpatient clinical and echocardiographic follow-up needs to be scheduled every 3–6 months [98]. The use of serial CMR, usually every 2 years in these stages, can provide essential information in patient’s management, especially with LGE progression and LVWT reduction, considering their predictive power in terms of EF reduction, HF occurrence and SCD. At this stage, a combination of cardiopulmonary exercise test (CPET) and echocardiography can be used to better define the prognosis, as described below (Table 1).

A summary of multimodality imaging findings at each HCM stage is shown in Table 2.

### 3.3. Follow-Up in the Advanced Stages: A Role for Instrumental Examinations?

There are not specific criteria to define advanced heart failure (AdvHF) in HCM. However, referral to a transplant center ought to be considered in a timely manner when high-risk features occur, due to possible rapid deterioration. Progression to AdvHF occurs in 3.5 to 17% of patients with HCM, and patients with sarcomere protein disease have higher probability to develop end-stage disease with LV systolic dysfunction compared to patients without mutations [99,100]. Timing between diagnosis of HCM and end-stage HCM has been reported to be between 4 and 10 years; conversely, only 2 to 3 years can pass from end-stage HCM onset to death or transplantation [101,102].

AdvHF in HCM is characterized by an increase in arrhythmic burden and/or decline in functional capacity. According to the 2020 AHA Guidelines, in patients with recurrent poorly tolerated life-threatening ventricular tachycardia, refractory to maximal anti-arrhythmic therapy and ablation, heart transplantation assessment is indicated with class I recommendation [3]. In addition, regardless of the LV EF, patients who experience severe and persistent symptoms of HF (NYHA class III or IV) despite guideline-directed treatment (pharmacological and septal reduction therapy) should be referred to a transplant center [2,3].

Reduced exercise capacity and progression to NYHA class III/IV is multifactorial and reflects different mechanisms. It is mainly due to the inability to increase the stroke volume secondary to diastolic dysfunction, chronotropic incompetence and severe LV obstruction. Interestingly, Maron et al. described a correlation between obstruction and rate of HF symptoms progression [34]. However, the exercise limitation can also follow adverse LV remodeling with diffuse replacement scarring, wall thinning, and systolic dysfunction [30,103]. In case of the presence of HF symptoms, CPET plays a relevant role in risk stratification and indication for AdvHF therapies. According to the International Society of Heart and Lung Transplantation Guidelines, listing criteria for heart transplant in the setting of HCM are similar to those for other cardiomyopathies. Peak VO2 ≤ 14 mL/kg/min or ≤50 % of predicted value in patents younger than 50 years of age are strongly associated with HF mortality [104]. In the presence of submaximal tests (RER < 1.05), the use of ventilation equivalent to a carbon dioxide slope (VE/VCO2) > 35 may be considered. In addition, several authors investigated correlations between CPET metabolic parameters and HF progression in the HCM population as summarized in Table 3 [105,106,107,108,109,110,111].

However, it is important to note that the populations considered were heterogeneous, including patients with obstructive and non-obstructive patterns, and the authors mainly investigated composite endpoints. Therefore, it is difficult to extrapolate binary prognostic markers to apply during routine clinical practice. Peak oxygen consumption alone does not fully reflect the complex physiopathology of advanced stages in HCM. Although useful to refer patients to specialized centers, decision to pursue heart transplantation should be based on the broad clinical assessment beyond CPET results.

On a different note, a combined approach of CPET and echocardiography enables evaluating the presence of exercise induced pulmonary arterial hypertension (EiPAH), that represents an important determinant of functional limitation in HCM patients. EiPAH was present in about one fifth of HCM patients without evidence of elevated pulmonary pressures at rest and was associated with adverse clinical outcome. Diagnosing EiPAH by exercise echo/CPET may help physicians to detect early stage of PAH requiring a closer clinical monitoring and individualized treatment strategies. Moreover, close vigilance for pulmonary hypertension (PH) is recommended in those patients who may become transplant candidates. PH is a potentially reversible contraindication for heart transplant usually treated with vasodilators, inotropes and/or durable left ventricle assist device (LVAD). Although LVAD implant as bridge to candidacy has been described in non-obstructive HCM, the majority of these patients is not suitable due to small LV cavity dimensions and severely impaired filling [112,113]. Therefore, referral to transplant centers should be made before significant PH is established. If treated with heart transplant, outcomes for HCM patients are similar or more favorable compared to other cardiomyopathies due to younger age and fewer co-morbidities at the time of transplant [114].

## 4. Future Perspectives: The Role of Artificial Intelligence

Artificial intelligence (AI) is described as “the theory and development of computer systems that can do activities that would ordinarily require human intellect, such as visual perception, speech recognition, decision-making, and language translation”. AI has been playing an increasingly important role in cardiology as a result of considerable advancements in information and communication technology, such as the straightforward storage, acquisition, and retrieval of vast amounts of data and knowledge [115].

AI has been increasingly used in HCM diagnosis, SCD prediction and treatment. Indeed, the multifactorial etiology of HCM, which includes structural and functional abnormalities, the burden of SCD in young adults with HCM and the severe morbidity in all age groups complicate diagnosis and treatment [116,117].

Since electrocardiographic abnormalities occur in more than 90% of HCM patients, 12-lead ECG may be a promising non-invasive, affordable, and quick method of screening [118]. However, ECG screening is constrained by significant false-positive rates due to the non-specific nature of the associated ECG features [119].

The use of an AI-based deep learning network has the potential to overcome these obstacles because it is trained to detect even the most subtle ECG patterns associated with structural changes, that may be undetectable by the human eye or traditional automated algorithms. Ko and co-workers developed an artificial intelligence approach for the detection of HCM based on ECG using a convolutional neural network (CNN) trained and validated on 2448 HCM patients and 51,153 non-HCM age- and sex-matched control subjects [120]. Overall, the AI algorithm demonstrated a high diagnostic performance with an AUC of 96%.

Concerning SCD prediction, the guidelines-recommended HCM Risk-SCD prediction model for guiding ICD implantation has a C-index of approximately 0.69, illustrating how fragile these methods are in the face of new data and how inadequately they perform [95]. The use of AI based classifiers outperformed the above-mentioned method and it also revealed 12 new predictors of ventricular arrhythmias [121].

Additionally, scar quantification by LGE-CMR, that may help in SCD risk stratification as previously reported, frequently relies on subjective, difficult, and time-consuming human demarcation of the myocardial borders and the hyperenhanced regions on LGE images [122]. The application of CNN to automatically measure left ventricle mass and scar volume on LGE showed excellent results with efficiency, as automatic segmentation was accomplished in 0.26 s/image [123].

In recent years, even if the great development of imaging technique and genetics have made significant contributions to our understanding of HCM, a more comprehensive approach incorporating broad-based testing across multiple ‘omics’ (e.g., proteomics, metabolomics, and genomics) may allow for improved precision in monitoring health and disease [124]. To date, machine learning technologies can efficiently manage this large amount of data to produce personalized physiological “portraits” of HCM patients, guiding precision approaches to therapy.

Among the obstacles to the development of machine learning is the need for a large dataset as a reference point for interpretation. This raises ethical concerns around the usage of patient data and the machines’ ability to maintain data security and confidentiality.

## 5. Conclusions

While the continuous advances in imaging techniques offer superb opportunities for a comprehensive evaluation of patients with HCM, only a profound knowledge of the specific mechanisms and patterns of disease will allow the appropriate use of these resources in each clinical setting of HCM. The comprehensive analysis of imaging data coupled with the potential of artificial intelligence is crucial to assessing the differential diagnosis with phenocopies, maximizing clinical management and achieving therapeutic benefits for specific needs at each stage of HCM. Promptly identifying patients at risk of developing advanced LV dysfunction and HF may drive preventive strategies over a period of several years. Disease progression towards HF, LVOT obstruction and risk of SCD may change over time: close clinical and well-scheduled imaging surveillance may potentially allow preventive treatment.

## Figures and Tables

**Table 1:** Timing for exam repetition: proposal of a practical approach for each disease stage.

Non-Hypertrophic Phase
ETT: every 2 years in carriers for a mutation associated with the development of HCM. First-degree relatives: every 1–2 years between 10 and 20 years old, and every 2–5 years in adults. CMR: in case of suboptimal/borderline echo images, high-risk families, ECG-positive/ETT-negative patients.
Classic phenotype
ETT: every year. Stress echocardiography: every 6 months in obstructive HCM, every 2 years in remaining patients. CMR: every 3 to 5 years.
Adverse remodeling and overt dysfunction
ETT: every 6 months or sooner (based on clinical status). CPET-ETT combination: every 6 months to 1 year (based on clinical status and before referral to a heart transplantation center). CMR: every 2 years.

TTE transthoracic echocardiography, CMR cardiac magnetic resonance, HCM hypertrophic cardiomyopathy.

**Table 2:** Abnormal findings at multimodality imaging in each HCM stage.

Non-hypertrophic	Imaging tests may be normal. Abnormal findings may include a reduction in e’ or abnormalities in myocardial strain + other non-specific patterns *
Classic phenotype	EF ≥ 65%, frequently abnormal LV strain frequent LVOTO normal diastole or delayed relaxation with reduced e’ mild-moderate LA dilation LGE: absent or <5% + other non-specific patterns *
Adverse remodeling	EF 50–65% less frequent LVOTO (a previously significant LVOTO may disappear) pseudonormal-restrictive diastole with reduced e’ moderate to severe LA dilation LGE 10–15% + other non-specific patterns *
Overt dysfunction	EF < 50% no LVOTO pseudonormal-restrictive diastole with severely reduced e’ severe biatrial dilation LGE 25–50% + other non-specific patterns *

* Other non-specific findings include abnormalities in papillary muscles and mitral leaflets and chordae, myocardial crypts, loss of progressive wall thinning from base to apex. EF, ejection fraction; LV, left ventricle; LVOTO, left ventricular outflow tract obstruction; LA, left atrium; LGE, late gadolinium enhancement.

**Table 3:** Papers focusing on the correlation between CPET metabolic parameters and HF progression in HCM.

First Author (Ref)	Year	Patients, N	End Point	CPET Prognostic Value
Sorajja et al. [105]	2012	182	HF progression, death	Peak VO2 < 60% predicted
Finocchiaro et al. [106]	2015	156	All-cause mortality, heart transplant, deterioration to septal reduction	VE/VCO2 > 34
Masri et al. [107]	2015	1005	All-cause mortality, sudden cardiac death	Peak VO2 < 50% predicted
Coats et al. [108]	2015	1898	All-cause mortality, heart transplant	Reduction of 21% of mortality/HT risk for each 1 mL/kg/min increase in peak VO2 and by 29% for each 1 mL/kg/min increase in AT. Risk of mortality/HT is decreased by 18% for each unit increase in VE/VCO2 slope
Magri et al. [111]	2016	623	Sudden cardiac death	VE/VCO2 slope > 31
Magri et al. [110]	2016	620	HF progression	Circulatory power (predicted peak VO2% peak SBP), VE/VCO2 slope (Mean peak VO2 = 21 mL/kg/min, mean VE/VCO2 slope = 29)
Magri et al. [109]	2018	681	HF events, sudden cardiac death	Peak HR < 70%

CPET, cardiopulmonary exercise test; HF, heart failure; HT, heart transplant; AT, anaerobic threshold; HR, heart rate.

## Supplementary Materials

The following supporting information can be downloaded at: https://www.mdpi.com/article/10.3390/life13010171/s1, File S1.
