# Supplementary material for: Multimodality Imaging in Sarcomeric Hypertrophic Cardiomyopathy: Get It Right…on Time
Source: Life (Basel). 2023 Jan 6;13(1):171. doi: 10.3390/life13010171 (PMC9863627; doi:10.3390/life13010171)
Supplement: Supplementary file 1 [file life-13-00171-s001.zip › life-2069570-supplementary.pdf]

Supplementary material

Methods

A literature search of MEDLINE/Pubmed library was performed for human studies in English language using the search terms “hypertrophic cardiomyopathy” and “imaging”. Further specific search terms focused on the single topics of “hypertrophic cardiomyopathy” and “echocardiography”, “stress echocardiography”, “cardiac magnetic resonance”, “phenocopies”, “advanced heart failure”, “cardiopulmonary exercise test” and “artificial intelligence”. Results were restricted to articles published in the last 5 years, to select the latest novelties in the field. Potentially pertinent articles were reviewed to exclude duplicates and to confirm the relevance in the field. The review included meta-analyses, clinical trials, randomized controlled trials, observational studies, and systematic reviews. Furthermore, the most relevant papers quoted in the selected articles and in more recent guidelines on this topic were included and bibliographies of identified studies were manually screened as well as potentially fitting papers. Among a total of 542 articles screened, at the end, 124 articles were finally selected in this review and appraised as full text.
